# A Physiologically Based Pharmacokinetic Model of Isoniazid and Its Application in Individualizing Tuberculosis Chemotherapy

**DOI:** 10.1128/AAC.00508-16

**Published:** 2016-09-23

**Authors:** Henrik Cordes, Christoph Thiel, Hélène E. Aschmann, Vanessa Baier, Lars M. Blank, Lars Kuepfer

**Affiliations:** Institute of Applied Microbiology (iAMB), Aachen Biology and Biotechnology (ABBt), RWTH Aachen University, Aachen, Germany

## Abstract

Due to its high early bactericidal activity, isoniazid (INH) plays an essential role in tuberculosis treatment. Genetic polymorphisms of *N*-acetyltransferase type 2 (NAT2) cause a trimodal distribution of INH pharmacokinetics in slow, intermediate, and fast acetylators. The success of INH-based chemotherapy is associated with acetylator and patient health status. Still, a standard dose recommended by the FDA is administered regardless of acetylator type or immune status, even though adverse effects occur in 5 to 33% of all patients. Slow acetylators have a higher risk of development of drug-induced toxicity, while fast acetylators and immune-deficient patients face lower treatment success rates. To mechanistically assess the trade-off between toxicity and efficacy, we developed a physiologically based pharmacokinetic (PBPK) model describing the NAT2-dependent pharmacokinetics of INH and its metabolites. We combined the PBPK model with a pharmacodynamic (PD) model of antimycobacterial drug effects in the lungs. The resulting PBPK/PD model allowed the simultaneous simulation of treatment efficacies at the site of infection and exposure to toxic metabolites in off-target organs. Subsequently, we evaluated various INH dosing regimens in NAT2-specific immunocompetent and immune-deficient virtual populations. Our results suggest the need for acetylator-specific dose adjustments for optimal treatment outcomes. A reduced dose for slow acetylators substantially lowers the exposure to toxic metabolites and thereby the risk of adverse events, while it maintains sufficient treatment efficacies. Vice versa, intermediate and fast acetylators benefit from increased INH doses and a switch to a twice-daily administration schedule. Our analysis outlines how PBPK/PD modeling may be used to design and individualize treatment regimens.

## INTRODUCTION

Although tuberculosis is a curable disease, about 9.6 million individuals still fell ill with tuberculosis and about 1.5 million people died from tuberculosis in 2014 (1). The World Health Organization (WHO) estimates that about one-third of the world's population is latently infected with Mycobacterium tuberculosis and the lifetime risk of developing active tuberculosis lies between 5 and 15% (2). Tuberculosis is caused by the inhalation of M. tuberculosis bacteria via airborne droplets spread by diseased individuals. The pathogen reaches the alveoli and distal airways in the lung of the host, where it proliferates and infiltrates tissue. Macrophages in the alveolar and interstitial space in the lung ingested the bacteria, initiating a cascade of events resulting in either successful containment of the infection or progression to active disease. An impaired immune system, such as that resulting from HIV infection or the intake of immune-suppressive substances, increases the susceptibility to the development of tuberculosis (3).

**FIG 1:**
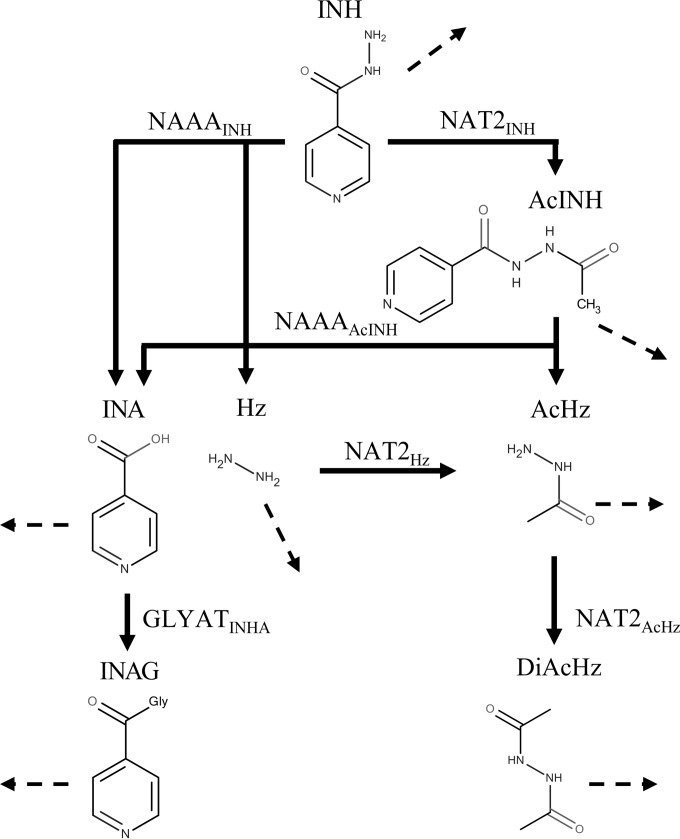
Isoniazid metabolism modeled in humans (67, 68), including enzymatic reactions (solid lines), renal excretion reactions (dashed lines), and transport reactions (dotted lines).

WHO recommends isoniazid (INH) as standard treatment for tuberculosis either as a single agent, such as for prevention therapy for latent tuberculosis and in HIV-infected individuals, or as a comedication together with rifampin, pyrazinamide, and ethambutol for the treatment of active pulmonary tuberculosis (2, 4). Isoniazid is an antibiotic specific for M. tuberculosis and among the first-line drugs used for the treatment of tuberculosis shows the greatest early bactericidal activity (EBA) (5–7), which is usually measured as the average decline in the log number of CFU (log_10_ number of CFU) in patient sputum samples during the first days of treatment (8). In pulmonary tuberculosis, INH exposure in the lungs determines the desired antimycobacterial activity. Previous studies showed that low plasma INH concentrations negatively affect treatment outcomes (9) and lead to longer treatment response times, higher rates of treatment failure, and the emergence of drug resistance (10, 11). Clinical observations linked patient susceptibility to the trimodal pharmacokinetics (PK) of INH, caused by the genetic polymorphisms of *N*-acetyltransferase type 2 (NAT2) (12). Although various NAT2 polymorphisms are known, patients can be categorized according to the number of functional NAT2 alleles that they have into slow, intermediate, and fast acetylator phenotypes (13). Besides acetylation, humans metabolize INH into various compounds, some of which are known to be toxic (14) (Fig. 1). Since the complex metabolism of INH is mainly dependent on NAT2 pharmacogenomics, NAT2 polymorphisms alter the PK of the parent drug, INH, as well as the PK of downstream metabolites, including the toxic compounds hydrazine (Hz) and acetylhydrazine (AcHz).

While the therapeutic effect of INH is undisputed, adverse effects during INH therapy occur in 5 to 33% of all patients receiving standard oral INH treatment at 300 mg once a day (QD) (15). These adverse events emerge from exposure to INH (16) and in particular to its toxic metabolites, Hz and AcHz, in liver and brain (17–19). Notably, both toxic metabolites are a substrate for NAT2 (20); thus, the enzyme is involved in both metabolite formation and subsequent detoxification. This results in an inevitable trade-off between treatment efficacy and drug-induced toxicity in INH-based chemotherapy. Although slow acetylators have an increased risk of adverse reactions due to a higher exposure to toxic metabolites (21, 22) and fast acetylators, in turn, have to face reduced treatment efficacies as a result of the lower plasma half-life of the active drug (23, 24), patients still receive the same INH doses, regardless of their acetylator or health status.

**FIG 2:**
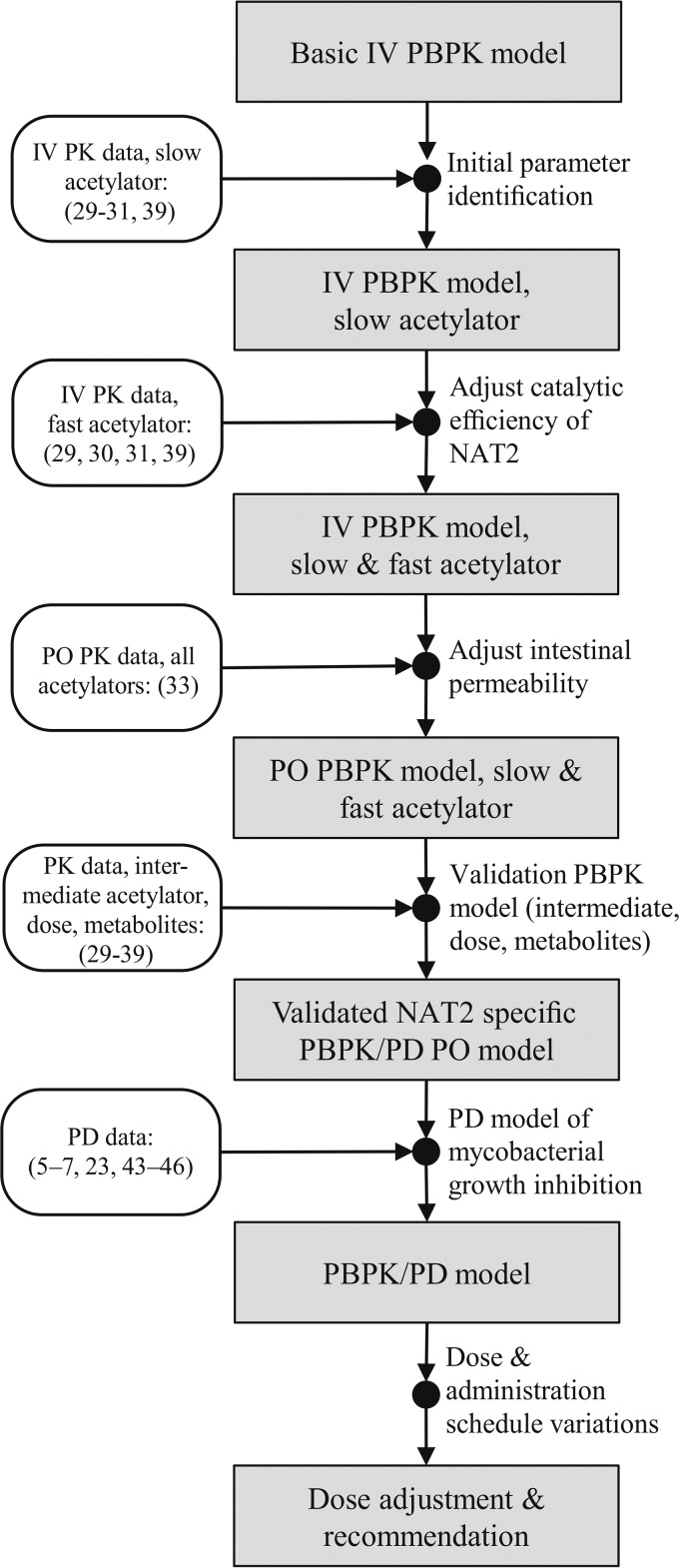
Work flow of PBPK/PD model development. The references used for model establishment and validation at the various steps are indicated. IV, intravenous administration; PO, oral administration.

In the study described here, we used NAT2 acetylator-specific physiologically based pharmacokinetic (PBPK) models of INH and its metabolites to investigate the trade-off between treatment efficacy and toxicity in INH-based chemotherapy. NAT2-specific PBPK simulations were performed on a population scale to analyze various INH doses and treatment schedules with regard to the risk-benefit ratios to be expected. PBPK models are in particular well suited for mechanistic analyses, since different organs are explicitly represented, allowing, among other things, estimation of concentration-time profiles in various tissues (25). In our analysis, on-target INH exposure in the interstitial space of the lung as well as intracellular exposure to the toxic metabolites Hz and AcHz in off-target organs, such as the liver, were simultaneously quantified. The established PBPK models were coupled with a pharmacodynamic (PD) model (Fig. 2), accounting for the antimycobacterial effect of INH on M. tuberculosis propagation in human lungs. The resulting PBPK/PD model was used to systematically evaluate different INH dosing schedules for NAT2-specific patient populations with a normal or an impaired immune system. Based on our simulation results, we provide suggestions for adjusted patient-specific dosing regimens that simultaneously take into account the patients' drug susceptibility and immune status.

## MATERIALS AND METHODS

### Isoniazid PBPK model.

An overview of the INH metabolism modeled is shown in Fig. 1. INH is mainly acetylated by NAT2 (NAT2_INH_) to acetylisoniazid (AcINH). AcINH is then hydrolyzed by an unknown acetylisoniazid hydrolase into isonicotinic acid (INA) and acetylhydrazine (AcHz). Here, we defined *N*-acylethanolamine acid amidase (NAAA) to be the catalyst for this metabolization step (NAAA_AcINH_). INA is conjugated to glycine by an unknown transferase to form isonicotinylglycine (INAG). On the basis of the chemical and structural similarity of INAG and hippuric acid, glycine-*N*-acyltransferase (GLYAT_INA_) was assumed to catalyze this reaction (26, 27). Due to its broad substrate spectrum (28), the basolateral T-type amino acid transporter (SLC16A10) was used for active INA and INAG transport (SLC16A10_INA_ and SLC16A10_INAG_, respectively). Besides the acetylation pathway, INH is directly converted into INA and hydrazine (Hz) by an unknown INH hydrolase. Here, we also assumed that NAAA catalyzed this reaction (NAAA_INH_), since the molecular site of the reaction is the same as that in the hydrolysis of AcINH to INA and AcHz. Hydrazine is acetylated by NAT2 (NAT2_Hz_) to AcHz, which is further acetylated by NAT2 to diacetylhydrazine (DiAcHz) (29).

The PBPK model comprising INH and its metabolites (AcINH, INA, INAG, AcHz, DiAcHz, and Hz), their metabolic reactions (NAT2_INH_, NAT2_Hz_, NAT2_AcHz_, NAAA_INH_, NAAA_AcINH_, GLYAT_INA_), active transport reactions (SLC16A10_INA_, SLC16A10_INAG_), and urinary excretion reactions (INH, AcINH, INA, INAG, Hz, AcHz, and DiAcHz) was built using the PBPK modeling software PK-Sim (version 6.0.3; Bayer Technology Services GmbH, Leverkusen, Germany). Model parameter identification and population simulations were conducted in MATLAB software (version 8.5.0.197613; The MathWorks, Inc., Natick, MA) by use of the MoBi toolbox for MATLAB (version 6.0.3; Bayer Technology Services GmbH, Leverkusen, Germany). PK-Sim and MoBi are freely available for noncommercial academic use. PK data were extracted from original publications with the WebPlotDigitizer web-based tool (version 3.9; Ankit Rohatgi, Austin, TX, USA). The parameters provided in Tables 1 and 2 and in Table S1 in the supplemental material are sufficient to fully parameterize the PBPK models, such that all PK profiles of INH and its metabolites can be reproduced. The physicochemical properties (lipophilicity, water solubility, molecular weight, and pK_a_ values) of all modeled compounds were calculated with MarvinSketch software (version 15.11.30.0; ChemAxon Kft., Budapest, Hungary) and used to parameterize the basic distribution model in PK-Sim. The INH fraction unbound (*f_u_*) in blood was taken from the literature, while the unknown *f_u_* values of the downstream metabolites were estimated during model development (Table 1).

**TABLE 1:** Physicochemical parameters for INH and its metabolites

Compound	Mol wt (g · mol^−1^)^*a*^	log P^*a*^	*f_u_*	pK_a_/pK_b_^*a*^	Solubility (g · liter^−1^) at pH 7.4^*a*^
Isoniazid	137.14	−0.67	0.9^*b*^	13.61/2.36, 3.36	42.15
Acetylisoniazid	179.18	−0.9	0.7^*c*^	11.43/9.04, 3.19	25.51
Isonicotinic acid	123.11	0.4	0.95^*c*^	3.73/2.35	5,326
Isonicotinylglycine	180.16	−0.7	0.95^*c*^	1.21/2.76, 3.88	2,400
Hydrazine	32.05	−1.96	0.99^*c*^	−/5.4, 3.71	200
Acetylhydrazine	74.08	−1.33	0.98^*c*^	13.18/3.28	4,183
Diacetylhydrazine	116.12	−1.62	0.99^*c*^	12.14, 10.83/−	125.76

a Estimated; log P, partition-coefficient; pK_a_, acid dissociation constant; pK_b_, base dissociation constant.

b Taken from reference 69.

c Identified from parameter optimization.

**TABLE 2:** Kinetic parameters for metabolic reactions of INH PBPK model

Enzymatic reaction	*V*_max_ (μmol · liter^−1^ · min^−1^)				*K_m_* (μmol · liter^−1^)
	All subjects	Slow acetylators	Intermediate acetylators	Fast acetylators	
NAT2_INH_		50	225	400	1,950
NAT2_Hz_		6	27	48	320
NAT2_AcHz_		0.5	2.25	4	10
NAAA_INH_	4				2,000
NAAA_AcINH_	3				500
GLYAT_INA_	0.25				10
SLC16A10_INA_	15				300
SLC16A10_INAG_	1				10

### Data collection and generation of patient populations.

PK data from healthy volunteers and tuberculosis patients from different clinical studies (Table 3) were used for model development and validation. Since no comprehensive PK data set for both INH and all its considered metabolites was available in the literature, we combined data sets from several studies (30–32) and initially identified a set of kinetic and compound parameters for slow acetylators (Tables 1 and 2 and Table S1 in the supplemental material). Further, data from an oral administration study were used to identify the intestinal absorption of INH (33). All other PK data considered (29, 34–39) were used for subsequent model validation. For each PK data set, patient anatomical and physiological characteristics as well as the study design, including dose and administration intervals, were specifically considered in the corresponding PBPK model.

**TABLE 3:** Human PK studies considered for model establishment and validation

Route^*a*^	Dose	Genotype^*b*^	Measured biomarker(s)	Sample	Reference	Use^*c*^
i.v.	8.38, 9.87 mg · kg^−1^	FF, SS	INH, AcINH, INA, INAG	Blood, urine	30	E/V
i.v.	5 mg · kg^−1^	FF, FS, SS	INH, AcINH, INA, INAG	Blood, urine	31	E
i.v.	10 mg · kg^−1^	SS	INH, AcINH, AcHz, DiAcHz	Blood	32	E
p.o.	300 mg	FF, FS, SS	INH, AcINH	Blood	33	E/V
p.o.	300, 600, 900 mg	FF, FS	INH	Blood	34	V
p.o.	4.75, 4.68 mg · kg^−1^	FF, SS	INH, Hz	Blood	35	V
p.o.	300 mg	FF, SS	INH, AcHz	Blood	36	V
p.o.	300 mg	FF, SS	AcHz, DiAcHz	Blood	37	V
p.o.	300 mg	FF, SS	INH, AcINH, AzHz, DiAcHz	Blood	38	V
p.o.	5 mg · kg^−1^	FF, SS	INH, AcINH, INA, INAG	Urine	39	V
p.o.	20 mg · kg^−1^	FF, SS	INH, AcINH, INA, INAG, AcINH, DiAcHz	Urine	29	V

a i.v., intravenous; p.o., oral.

b FF, fast acetylator; FS, intermediate acetylator; SS, slow acetylator.

c E, establishment; V, validation.

For population simulations, mean patient PBPK models were used. A virtual population of a mean PBPK model was created by varying anatomical and physiological parameters (40) for 1,000 individuals (see Table S2 in the supplemental material).

### Pharmacodynamics of isoniazid against M. tuberculosis.

The PD model describes the change in mycobacterial growth, due to the exposure to INH in the interstitial space of the lung with an additional contribution of the immune system (equation 1).

(1) $$\frac{dN(t)}{dt}=N_{0}\cdot [\text{μ}\;-\;\beta_{0}\;-\gamma (C,\;E_{\text{max}},\text{MIC},Km)]$$

where *N* is the number of bacteria, *t* is time, *N*_0_ is the initial bacterial load of M. tuberculosis, *C* is the time-dependent concentration of unbound INH in the interstitial space of the lung obtained from PBPK model simulations, *E*_max_ is the maximal antimicrobial effect of INH, MIC is the MIC of INH taken from the literature (41) and represents a threshold for the INH concentration needed to achieve an antimycobacterial effect, and *K_m_* is the INH concentration at which half the maximal antimicrobial effect is reached. To obtain the uninhibited mycobacterial growth in humans (μ), the growth rate of M. tuberculosis in sputum derived from untreated tuberculosis patients (23) was multiplied by the growth rate ratio in wild-type mice (immunocompetent) and immune-deficient mice infected with M. tuberculosis via the tail vein (42), therefore accounting for the immune response of the host. The contribution of the immune system to antimycobacterial inhibition (β_0_) was estimated during the exponential growth phase in *in vivo* experiments in mice and humans (5–7, 23, 42–46). A detailed description of the calculation of μ and β_0_ can be found in the supplemental material. The INH-induced killing (γ) was modeled as a previously described inhibitory sigmoidal *E*_max_ model (47), given by equation 2:

(2) $$\gamma (E_{\text{max}},C,\text{MIC},Km)=\frac{E_{\text{max}}\cdot {\left(C/\text{MIC}\right)}^{h}}{Km^{h}+{\left(C/\text{MIC}\right)}^{h}}$$

where *h* is Hill's constant describing the sigmoidicity of the inhibition curve and the other terms are as defined above. Coupling of the two model equations, equation 2 and equation 1, yields

(3) $$\frac{dN(t)}{dt}=N_{0}\cdot \;\left[\text{μ}-\beta_{0}\;-\frac{E_{\text{max}}\cdot {\left(\text{C}/\text{MIC}\right)}^{h}}{Km^{h}+{\left(C/\text{MIC}\right)}^{h}}\right]$$

The pharmacodynamics of immunocompetent and immune-deficient patients can be simulated with equation 3. To account for immune deficiency, β_0_ is replaced by β* = β_0_ · *d*_,_ where *d* accounts for the strength of the immune response in an individual (the value of *d* ranges from 1 for a fully immunocompetent individual to 0 for a fully immune-deficient individual). This allows the various stages of severity of immune deficiency, such as those encountered during the progression of HIV infection, to gradually be described. Immune-deficient populations were generated by sampling *d* from a uniform distribution to equally account for the various severity stages. The parameters of *d* used for the immune-deficient populations can be found in Table S3 in the supplemental material.

### Clinical indices.

Clinical indices were used to describe treatment efficacy (*E*) at the site of infection in the lungs and the toxicity (*T*) induced in the off-target organ evaluated, the liver, due to the exposure to toxic metabolites. Both were calculated for each individual in all populations relative to benchmarks, which were the treatment efficacy and toxicity for a corresponding individual with a slow acetylation phenotype receiving the recommended standard regimen of 300 mg INH QD over 2 days of treatment (100%).

For each individual *i* in a virtual population of genotype *g* receiving dose *d* in administration interval *a*, the EBA was estimated from the PBPK/PD model by equation 3. The treatment efficacy relative to that for the same individual *i* (*E_i_*) who was a slow acetylator receiving the standard dose of 300 mg INH in a QD dosing regimen was than derived by equation 4:

(4) $$E_{i}=\frac{{\text{EBA}}_{i}^{d}ag}{{\text{EBA}}_{i}^{300\text{mg}}\text{QD}\text{slow}}$$

Analogously to treatment efficacy, the toxicity index for individual *i* (*T_i_*) was calculated for each individual in all virtual populations by equation 5. The toxicity index is a measure of the toxic events experienced due to exposure to the toxic metabolites Hz and AcHz. Exposure to the two toxic metabolites Hz and AcHz was defined as the area under the concentration-time curve (AUC) in the intracellular space in the off-target organ, the liver, and was estimated from the PBPK model.

(5) $$T_{i}=\frac{\left[\text{AUC}{\left(\text{Hz}\right)}_{i}^{d}ag+\text{AUC}{\left(\text{AcHz}\right)}_{i}^{d}ag\right]}{\left[\text{AUC}{\left(\text{Hz}\right)}_{i}^{300\text{mg}}\text{QD}\text{slow}+\text{AUC}{\left(\text{AcHz}\right)}_{i}^{300\text{mg}}\text{QD}\text{slow}\right]}$$

Both clinical indices can exceed 100%, if a combination of dose and acetylator phenotype results in a larger EBA after 2 days of treatment for treatment efficacy or higher exposure to toxic metabolites for the toxicity index. Negative values of treatment efficacy can occur if mycobacterial growth (μ) exceeds the antimycobacterial effects of immune-dependent killing (β_0_, β*) and INH-induced killing (γ).

## RESULTS

### PBPK model development.

**FIG 3:**
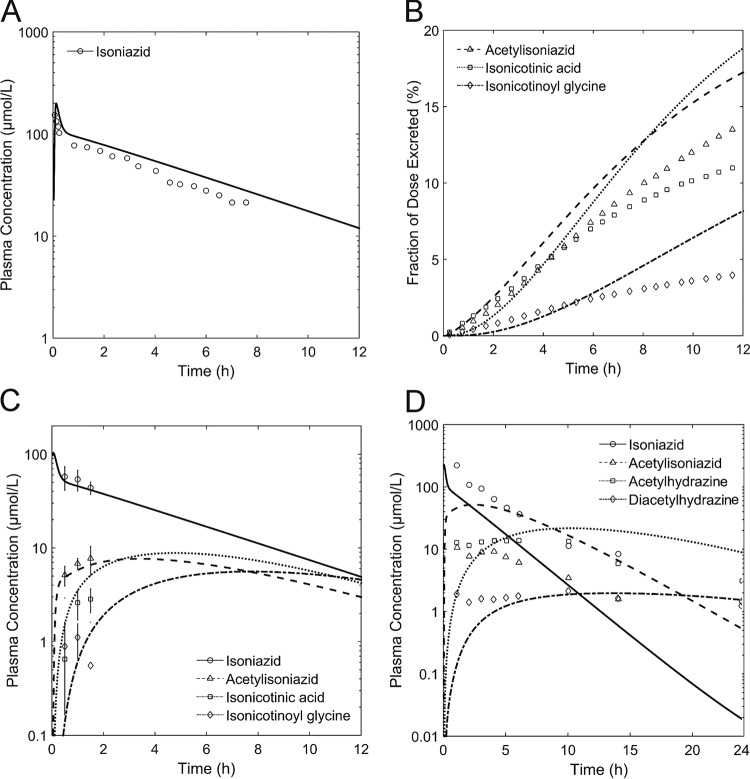
Results of simulation with the initial PBPK model for intravenously administered INH in slow acetylators. The plasma concentration profiles of INH and its metabolites are shown. Simulations are shown as lines, and experimental data are shown as symbols. (A) Simulated and observed (30) PK of INH; (B) simulated and observed (30) renal excretion as a fraction of the INH dose for AcINH, INA, and INAG; (C) simulated and observed (31) PK of INH, AcINH, INA, and INAG; (D) simulated and observed (32) PK of INH, AcINH, AcHz, and DiAcHz.

By following the model development work flow outlined in Fig. 2, a PBPK model of intravenously administered INH and its metabolites (AcINH, INA, INAG, AcHz, DiAcHz) for slow acetylators was first established (Fig. 3A to D). The metabolization reactions of INH and its metabolites (Fig. 1) were described by Michaelis-Menten kinetics. Since no comprehensive PK data set for INH and all of its metabolites has been published to date, data from several studies (30–32) were combined. Physicochemical parameters, such as lipophilicity, the fraction unbound, and molecular weight, were used to parameterize the basic distribution model (25). Fine-tuning of these parameters, as well as identification of the kinetic parameters in the Michaelis-Menten equations (*K_m_*, maximum rate of metabolism [*V*_max_]), was subsequently done by minimizing the error between the simulated and observed plasma concentration-time profiles and urinary excretion ratios of INH and its metabolites. Notably, all of the PK data used for model establishment were taken from the initial data set for slow acetylators comprising data on the PK profiles for single patients and population means. After parameter identification, the simulated PK profiles were in good agreement with the experimental data for slow acetylators (correlation coefficient [*R*^2^] = 0.58, *P* < 0.001) (Fig. 3A to D). All model parameters are given in Tables 1 and 2 and in Table S1 in the supplemental material.

Next, an model for intravenous administration in fast acetylators was developed on the basis of the initially identified set of parameters. Following earlier work (48), we hypothesized that polymorphisms in NAT2 change the catalytic enzyme activity and may be sufficient to explain the altered INH PK in the patients with the fast acetylator phenotype. Therefore, the catalytic activities (*V*_max_s) of NAT2-catalyzed reactions (Fig. 1) were changed by use of a constant factor, while all other model parameters were kept constant during the following analyses. We found that an 8-fold increase in NAT2 catalytic activity is sufficient to obtain accurate descriptions of the PK profiles of both INH and its metabolites (AcINH, INA, and INAG) in fast acetylators (*R*^2^ = 0.94, *P* < 0.001) (30, 31) (see Fig. S2A to C in the supplemental material). Hence, adjustment of a single parameter (*V*_max_) was enough to explain the differences in PK between slow and fast acetylators, as such, meeting the expectations from clinical practice.

**FIG 4:**
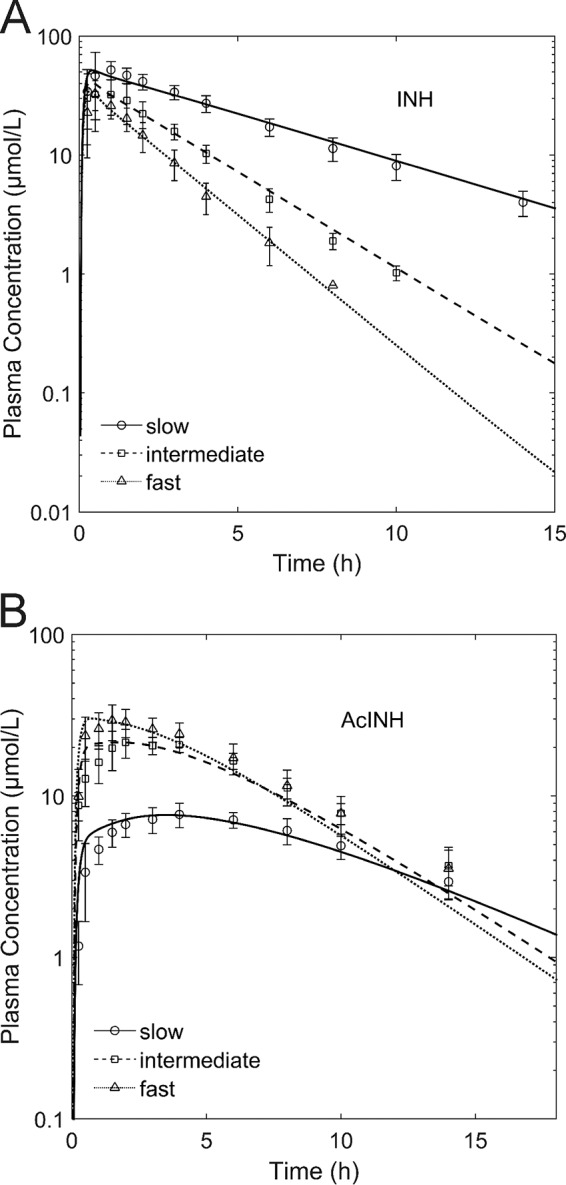
Validation of NAT2 phenotype-dependent INH (A) and AcINH (B) PK. Results of a simulation with a QD oral dose of 300 mg INH are shown as lines, and experimental data (50) are shown as symbols.

On the basis of all previously identified parameters in the PBPK model for intravenously administered INH, a PBPK model for orally administered INH for slow and fast acetylators was built. Only the intestinal permeation of INH was adjusted to account for the changed route of administration. The intestinal permeation parameter was found to be 1 × 10^−4^ cm · min^−1^, which is well within the range of experimentally determined values from *in vitro* studies (1.2 × 10^−6^ cm · min^−1^) (49) and *in vivo* studies (9.2 × 10^−4^ cm · min^−1^) (50) in rats. To validate our model, we next estimated the catalytic activity of intermediate acetylators in the models of oral treatment by using the arithmetic mean of the catalytic activity of slow and fast acetylators, as already demonstrated in a previous study for statin pharmacogenomics (48). The PK profile of the intermediate acetylator was hence a pure prediction. As for the previous PBPK models, we found a good correlation between the simulated and observed PK profiles (33) for INH (Fig. 4A) and AcINH (Fig. 4B) (*R*^2^ = 0.93, *P* < 0.001), indicating the structural correctness of the PBPK model with oral administration.

Next, the model with oral administration was further validated for different doses for fast (300 mg, 600 mg, 900 mg) and intermediate (300 mg) acetylators (34), which were not considered during the previous model validation steps. The simulated PK profiles of INH were in good agreement with the experimental data (*R*^2^ = 0.94, *P* < 0.001) (see Fig. S3 in the supplemental material), as such, further supporting the validity of the PBPK model of INH. Further, experimental PK data were used to additionally verify the metabolite PK predictions (29, 34–39) (Table 4; see also Fig. S5 to S8 in the supplemental material).

**TABLE 4:** PBPK model validation results

Route^*a*^	No. of subjects	Dose	NAT2^*b*^	Pearson correlation coefficient (RMSD^*c*^)							Reference
				INH	AcINH	INA	INAG	Hz	AcHz	DiAcHz	
i.v.	1	8.38 mg · kg^−1^	FF	0.99 (0.77)							30
i.v.	1	9.87 mg · kg^−1^	SS	0.98 (0.32)							30
i.v.	6	5 mg · kg^−1^	SS	0.82 (0.12)	0.99 (0.16)	0.82 (0.98)	0.19 (0.91)				31
i.v.	13	5 mg · kg^−1^	FS	0.95 (0.09)	0.96 (0.18)	0.72 (0.61)	−0.21 (0.66)				31
i.v.	2	5 mg · kg^−1^	FF	1 (0.03)	0.98 (0.12)	0.9 (0.55)	0.33 (0.7)				31
i.v.	1	10 mg · kg^−1^	SS	0.99 (0.73)	0.91 (4.34)				−0.04 (1.27)	0.02 (0.45)	32
p.o.	8	300 mg	SS	0.97 (0.14)	0.75 (0.54)						33
p.o.	8	300 mg	FS	0.98 (0.28)	0.79 (0.21)						33
p.o.	8	300 mg	FF	0.97 (0.18)	0.91 (0.34)						33
p.o.	8	300 mg	FS	0.99 (0.32)							34
p.o.	8	300 mg	FF	0.99 (2)							34
p.o.	8	600 mg	FF	1 (0.37)							
p.o.	8	900 mg	FF	0.97 (0.48)							
p.o.	11	4.75 mg · kg^−1^	SS	0.7 (0.35)				0.28 (0.83)			35
p.o.	15	4.68 mg · kg^−1^	FF	0.93 (0.37)				0.7 (0.63)			35
p.o.	3	300 mg	SS	0.83 (0.31)					0.7 (0.82)		36
p.o.	2	300 mg	FF	0.66 (0.52)					0.21 (0.61)		36
p.o.	3	300 mg	SS						0.71 (0.77)	0.85 (0.46)	37
p.o.	3	300 mg	FF						0.75 (0.89)	0.72 (0.77)	37
p.o.	1	300 mg	SS	0.94 (0.66)	−0.09 (0.96)				0.89 (0.6)	0.89 (0.83)	38
p.o.	1	300 mg	FF	0.98 (0.31)	−0.06 (1.87)				0.003 (0.66)	0.2 (0.73)	38

a i.v., intravenous; p.o., oral.

b FF, fast acetylator; FS, intermediate acetylator; SS, slow acetylator.

c RMSD, root mean square deviation.

### Population PBPK simulations.

Representative anatomical and physiological parameters of the PBPK models, such as organ weights, blood flow rates, and tissue compositions, were varied next (40) (see Fig. S2 in the supplemental material) to perform simulations with virtual populations of slow acetylators (see Fig. S4A and D in the supplemental material), intermediate acetylators (see Fig. S4B and E in the supplemental material), and fast acetylators (see Fig. S4C and F in the supplemental material). Notably, the model could correctly predict that the population median concentrations of INH and AcINH and the experimentally measured deviations were well within the simulated interquartile range.

### PBPK/PD model development.

**FIG 5:**
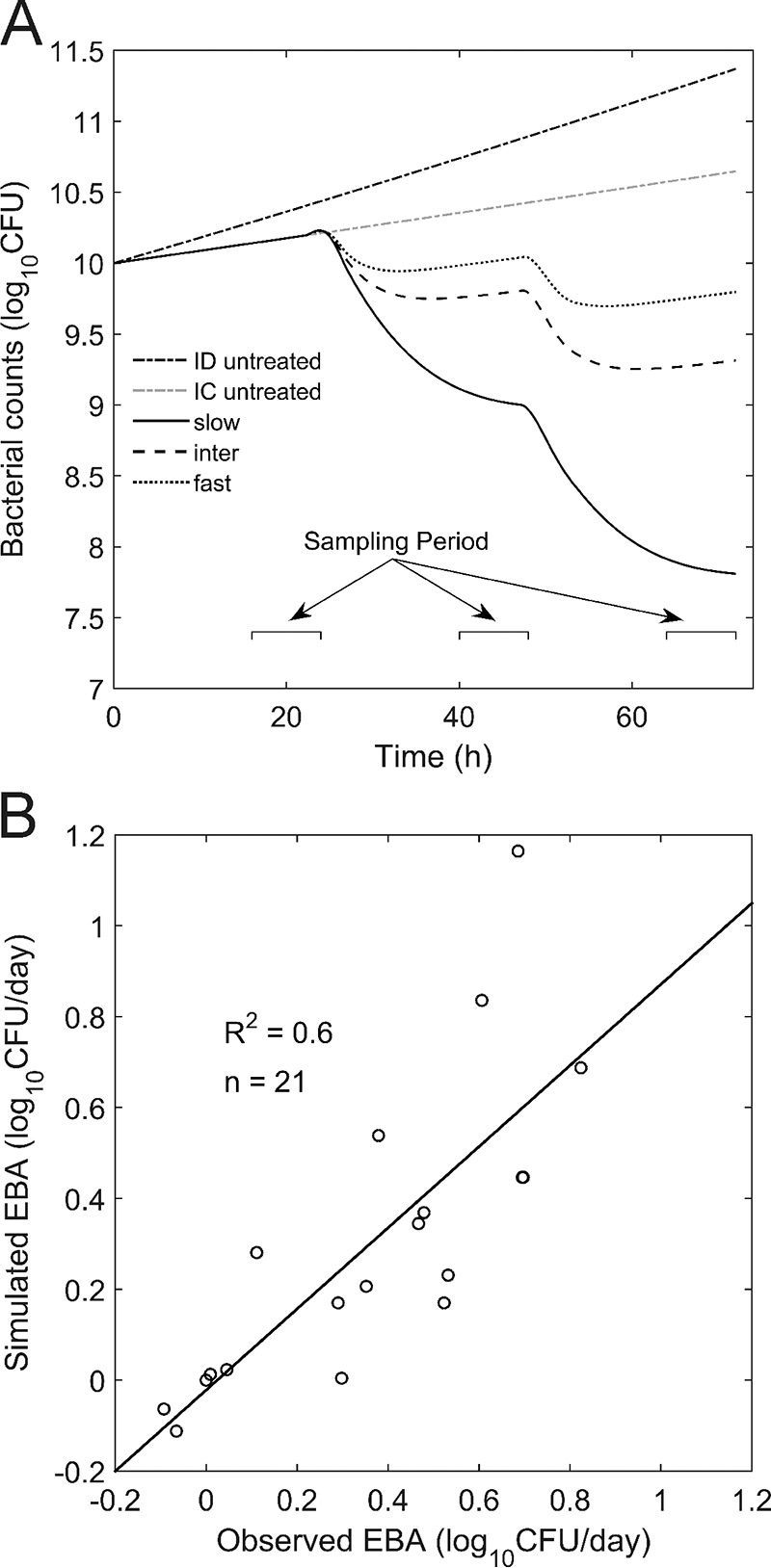
PD simulations. (A) Exemplary simulation of mycobacterial counts in human lungs following QD oral administration of 600 mg INH for 2 days of treatment. Simulation results for an untreated immune-deficient (ID) patient, an untreated immunocompetent (IC) patient, and an immunocompetent INH-treated patient are shown. (B) Observed (23, 45) versus predicted EBA after 2 days of INH chemotherapy for slow, intermediate, and fast acetylators receiving QD INH doses ranging from 9 mg to 600 mg.

Having established and validated a PBPK model for the description of INH pharmacokinetics, we next addressed the efficacy and toxicity of INH therapies. The PBPK model for oral administration was therefore coupled with a pharmacodynamic (PD) model of mycobacterial growth in human lungs to analyze the influence of the NAT2 phenotype, INH dose, and administration intervals on the efficacy and toxicity of INH-based tuberculosis treatment. The efficacy of INH for tuberculosis chemotherapy was the result of INH exposure in the human lungs, the site of infection. Simulated profiles of the concentration of unbound INH in the interstitial space of the lung, as such, representing on-target drug exposure, were used as the effective drug input for the PD model (equation 3). A sigmoidal *E*_max_ model was used (47) to describe the isoniazid-induced inhibition of mycobacterial growth. The model also takes the antibacterial contribution of the immune system into account (see Materials and Methods). The resulting PBPK/PD model allows simulation of the isoniazid-induced inhibition of mycobacterial growth in the interstitium of the lung of an infected patient. In particular, the effects of different cofactors, such as the administered dose, dosing schedules, NAT2 acetylator phenotype, and immune status, can thereby be simultaneously taken into account. The PD model parameters used are listed in Table 5. The interplay between PBPK and PD simulations with a dose of 600 mg INH QD is exemplarily shown in Fig. 5A. The predicted EBA in slow, intermediate, and fast acetylators receiving INH as a 600-mg, 300-mg, 150-mg, 75-mg, 37.5-mg, or 9-mg QD dosing regimen showed a good overall correlation with the observed data (*R*^2^ = 0.6, *P* < 0.001) (Fig. 5B).

**TABLE 5:** PBPK/PD model parameters

Parameter	Value	Reference(s) or source
*N*_0_	10 log_10_ CFU · liter^−1^	Arbitrary
μ	0.048 · day^−1^	(5, 6, 23, 43–46)^*a*^
β_0_	0.0219 · day^−1^	(42, 70)^*a*^
*E*_max_	0.534 · day^−1^	Fitted
*K_m_*	25.19 μmol · liter^−1^	Fitted
*h*	0.56	Fitted
MIC	1.46 μmol · liter^−1^	41

a A detailed calculation is provided in the supplemental material.

### Patient immune status.

The impact of different INH therapies in terms of the administered dose or treatment schedule can be immediately tested with the described PBPK/PD model. Different acetylator phenotypes can be explicitly considered, such that the therapeutic success to be expected can be specifically simulated. Patient immune status can also be easily taken into account, since the contribution of the host immune response to antimycobacterial activity is explicitly considered in the PD model (see Materials and Methods). Immune deficiency due to comorbidities, such as HIV infections or comedication with immune-suppressant drugs, is a potentially disadvantageous condition during isoniazid-based therapies for tuberculosis (3). In addition to immunocompetent individuals, we also considered immune-deficient patients in our analysis. For an immune-deficient individual, the antimicrobial inhibition mediated by the immune system is reduced. For the virtual population, simulations of immune-deficient individuals at various stages of immune deficiency were included to account for comorbidities (see Materials and Methods). In the following analysis, the impact of different daily INH doses (range, 9 mg to 1,200 mg) and two dosing schedules (QD and twice a day [BID]) on treatment efficacy for slow, intermediate, and fast acetylators in immunocompetent and immune-deficient populations was systematically analyzed.

### Clinical indices.

**FIG 6:**
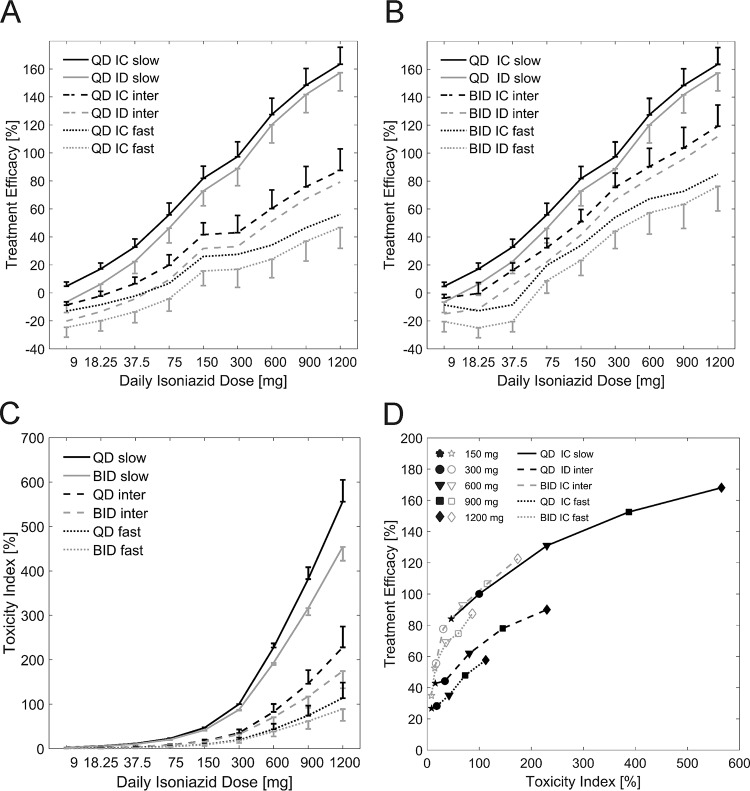
(A and B) Simulated treatment efficacy for immunocompetent (IC) and immune-deficient (ID) slow, intermediate (inter), and fast NAT2 acetylators receiving INH QD (A) and BID (every 12 h) (B). (C) Simulated toxicity index for slow, intermediate, and fast NAT2 acetylators receiving cumulative QD and BID INH doses ranging from 9 mg to 1,200 mg. Both treatment efficacy and the toxicity index were normalized to those for the slow acetylators receiving 300 mg INH QD. (D) Trade-off between treatment efficacy and toxicity index for INH doses of 1,200 mg, 900 mg, 600 mg, 300 mg, and 150 mg administered QD and BID.

An ideal therapy provides maximal treatment efficacy with minimal toxicity. In INH-based tuberculosis chemotherapy, known side effects, such as jaundice and peripheral neuropathy, occur in the liver and brain, respectively. These side effects are most likely caused by exposure to the toxic INH metabolites Hz and AcHz (17–19). To identify optimal INH dosing regimens for each NAT2 acetylator phenotype, we assessed the on-target efficacy (Fig. 6A and B) in the lungs and off-target toxicity (Fig. 6C) in the liver. On the basis of the findings, the resulting trade-off (Fig. 6D) in INH-based tuberculosis chemotherapy was assessed. The treatment efficacy of each dosing regimen relative to the treatment efficacy achieved in slow acetylators receiving the recommended regimen of an oral dose of 300 mg INH QD was calculated (equation 4). The toxicity index was considered analogously. Here, exposure (i.e., the AUC) to the toxic metabolites Hz and AcHz in the liver was used (equation 5).

### Treatment efficacy.

The treatment efficacy for each individual in the virtual population of immunocompetent and immune-deficient patients in the slow, intermediate, and fast acetylator groups was simulated for oral INH doses ranging from 9 mg to 1,200 mg. The INH doses administered to intermediate and fast acetylators were simulated as QD (Fig. 6A) and BID (Fig. 6B) regimens. For slow acetylators, BID administration was not considered since INH exposure was already the highest compared to that for the intermediate and fast acetylators. As expected, for all acetylator phenotypes, treatment efficacy increased with higher doses for doses ranging from 9 mg to 1,200 mg for both QD and BID administration (Fig. 6A and B), irrespective of patient immune status. In contrast to the treatment efficacy for slow acetylators, treatment efficacies were lower for the intermediate and fast acetylators in both the immunocompetent and immune-deficient populations. The average treatment efficacies for immune-deficient intermediate and fast acetylator populations receiving 300 mg INH QD were 44.2%, and 28.2%, respectively, compared to the treatment efficacy for the benchmark population, slow acetylators receiving 300 mg INH QD. Reduction of the administered dose to 150 mg QD maintained 84.1% of the original treatment efficacy in immunocompetent slow acetylators. The simulated treatment efficacy for intermediate and fast acetylators could be significantly increased when higher doses were considered, as such, compensating for the higher INH clearance in these subgroups. However, for intermediate and fast acetylators, even the highest tested INH dose of 1,200 mg in a QD regimen resulted in only 90% and 57.6% of the benchmark treatment efficacy, respectively. For immune-deficient populations, the treatment efficacies achieved were even lower: 81.4% and 48% of the benchmark treatment efficacy for intermediate and fast acetylators, respectively.

Besides higher INH dosages, increased treatment efficacy could be achieved by changing the administration schedule from QD to BID. A 600-mg INH BID regimen (300 mg 2 times a day, every 12 h) for an immunocompetent intermediate acetylator population resulted in 92.8% of the benchmark treatment efficacy. The 1,200-mg BID regimen achieved 87.4% of the benchmark treatment efficacy in fast acetylators in the immunocompetent population, while it achieved 84% and 78.4% of the benchmark treatment efficacy in intermediate and fast acetylators in the corresponding immune-deficient population, respectively.

Our PBPK/PD simulations showed that bisecting the daily INH dose for slow acetylators maintained 84.1% of the benchmark treatment efficacy. However, for an immune-deficient fast acetylator population, even a 4-fold increase of the QD INH dose up to 1,200 mg QD reached only 48% of the benchmark treatment efficacy. Notably, for this population, a switch from a 300-mg QD dosing regimen (which achieved 17.3% of the benchmark treatment efficacy) to a 300-mg BID dosing regimen (which achieved 45.4% of the benchmark treatment efficacy) had a larger impact on treatment efficacy than doubling of the administered QD dose to 600 mg (which achieved 24.7% of the benchmark treatment efficacy) (Fig. 6A and B).

### Toxicity index.

The toxicity index was simulated for INH doses ranging from 9 mg to 1,200 mg in slow, intermediate, and fast acetylator populations. Here, only immunocompetent patients were considered, since the immune status has no influence on INH PK (51). For intermediate and fast acetylators, the administered INH doses were simulated as QD and BID dosing regimens. Our simulations showed higher levels of exposure to the toxic metabolites Hz and AcHz in the intracellular liver compartment for slow acetylators than for intermediate and fast acetylators at all doses tested (Fig. 6C).

The average toxicity indices of the slow, intermediate, and fast acetylator populations receiving 300 mg INH QD were 100%, 34.2%, and 18.4%, respectively. Bisecting the standard regimen to 150 mg QD for slow acetylators resulted in less than half (46.4%) of the benchmark toxicity. Intermediate acetylators receiving 600 mg INH QD reached a toxicity index of 81.2%, while BID administration resulted in a toxicity index of 68.6%. In fast acetylators, 600 mg INH QD resulted in a toxicity index of 42.2%. The toxicity index of the highest tested dose of 1,200 mg INH QD was 112.7% of that for the benchmark population, while BID administration resulted in a toxicity index below the benchmark toxicity (86.8%). The switch to a BID administration scheme led to an overall reduction of the toxicity index for both intermediate and fast acetylators (Fig. 6C).

Changing the administration schedule from QD to BID for intermediate and fast acetylator populations allowed the toxicity index to be reduced such that the daily INH dose could be increased above 600 mg without exceeding the benchmark toxicity. For fast acetylators, the toxicity of doses even above doses of 900 mg QD and 1,200 mg BID did not exceed the benchmark toxicity. The identified dose and administration regimens for intermediate and fast acetylators resulted in treatment trade-offs (between efficacy and toxicity) equivalent to those for slow acetylators receiving the standard treatment (Fig. 6D).

## DISCUSSION

Several polymorphisms in *N*-acetyltransferase type 2 (NAT2) lead to altered catalytic activities for INH acetylation (52–55). As a consequence, the PK profiles of INH and its metabolites differ significantly between individuals. Patients can be categorized according to their number of functional NAT2 alleles into slow, intermediate, and fast acetylator phenotypes. Besides the parent drug, INH (20), the catalytic activity of NAT2 also affects the PK of the toxic metabolites Hz and AcHz (35, 37, 56, 57) (Fig. 1).

In the study described here, we developed a PBPK model for INH and six of its metabolites (AcINH, INA, INAG, Hz, AcHz, and DiAcHz). The model was validated with various sets of PK data, including those obtained by the use of intravenous and oral administration routes; different daily doses; different dosing schedules; and, most importantly, slow, intermediate, and fast acetylator phenotypes (Table 4).

The initial PBPK model was established for intravenous administration in slow acetylators. The kinetic parameters for INH metabolization and excretion were identified on the basis of a merged clinical data set (30–32) to allow the simultaneous consideration of INH and its metabolites, including data from single patients and population means. The different origins of the PK data sets from the literature used for model establishment might explain the deviations between the data from the simulations and the experimental data, as seen, for example, in the overestimated INH clearance in single patients (Fig. 3B and D). However, the use of a single set of parameters from different publications to describe PK data is a rather demanding approach to ensure model quality (Fig. 2). Furthermore, we carefully validated the overall structure of the PBPK model by prediction of PK in intermediate acetylators (Fig. 4) and by extrapolation to different doses (see Fig. S3 in the supplemental material). In the next step, a PD model describing the antibacterial activity of INH against M. tuberculosis was developed. The PK profiles of unbound INH in the interstitial space of the lungs were used as input for the mycobacterial growth model, thus coupling the PBPK model to the PD model. Previous studies showed that the INH concentrations in plasma, epithelial lining fluid, and alveolar cells do not differ significantly (58); therefore, we considered the unbound interstitial lung concentrations to be a reasonable approximation of the on-target availability of the therapeutic agent INH.

With the combined PBPK/PD model, we could show that the simulated treatment efficacies were consistent with experimental data describing the time-resolved EBA during the first 2 days of INH treatment. Clinical treatment durations continuing for months could not be analyzed with our model due to a lack of adequate experimental data. Likewise, the emergence of resistant mycobacterial subpopulations, as suggested by other authors (59, 60), was not considered. For our simulations, we chose a conservative estimate of the MIC of 0.2 mg · liter^−1^ to include most INH-susceptible M. tuberculosis strains (0.05 mg · liter^−1^ < MIC < 0.1 mg · liter^−1^ for most strains) (41). As a consequence, this high activity threshold causes the model to rather underestimate the EBA, which can be seen as an additional margin of safety for treatment and toxicity simulations.

We next performed population simulations to consider the effect of interindividual variability on the trade-off between treatment efficacy and drug-induced toxicity. To this end, virtual populations including 1,000 individuals each were generated for all acetylator types. The treatment efficacy and drug-induced toxicity of slow acetylators receiving the recommended standard therapy of 300 mg INH QD were set as benchmarks for both clinical indices for all other populations varying in their immune and acetylator status. This enabled in particular the identification of acetylator-specific dosing regimens, balancing the inherent trade-off between efficacy and toxicity during INH-based therapies. With the developed PBPK/PD model, the influence of the NAT2 acetylator phenotype, the administered dose, the immune status, and the dosing schedule on treatment efficacy and toxicity was systematically analyzed. Our simulation results suggest adjusted INH doses and administration regimens that may be used for immunocompetent and immune-deficient individuals of all acetylator phenotypes.

Slow acetylators receiving the standard regimen were considered the benchmark, since the greatest EBA is seen in individuals with this phenotype and such individuals have the greatest risk of experiencing drug-induced toxicities during INH-based tuberculosis therapies (61). We found that slow acetylators could benefit from reduced daily INH doses, since bisecting the standard dose to 150 mg QD would maintain high treatment efficacies and simultaneously reduce the exposure to the toxic metabolites Hz and AcHz in the off-target organ evaluated, the liver. For intermediate and fast acetylators, we found that the INH dose administered QD could be increased up to 600 mg and 900 mg, respectively, without the toxicity exceeding the benchmark toxicity. Furthermore, intermediate and fast acetylators could especially benefit from a modified administration regimen. Here, a switch to a BID administration schedule would allow dosing with INH at dose of up to 900 mg and 1,200 mg for intermediate and fast acetylators, respectively, without the toxicity exceeding the benchmark toxicity. The simulated treatment efficacy for intermediate and fast acetylators is consistent with previous findings in the literature showing that low plasma INH exposure increases the risk of experiencing treatment failure and relapse (9, 11). Our simulations also showed that a switch from a QD to a BID administration regimen resulted in greater treatment efficacies than doubling of the QD INH dose.

Irrespective of its clinical relevance, so far only a minority of clinical trials have accounted for the individual NAT2 pharmacogenomics of enrolled patients and their mycobacterial drug susceptibility. Our study provides mechanistic insights into tuberculosis treatment outcomes in individuals by taking the patient's pharmacogenomics and the pathogen's drug susceptibility into account. Moreover, our work provides a rational dosing design to balance the inherent trade-off between treatment efficacy and toxicity in INH-based chemotherapy. Previously, authors suggested an adaptation of administered INH dosages according to patient acetylator status (62–64). In a clinical trial, Azuma et al. modified a INH QD dose of 5 mg · kg^−1^ of body weight to doses of 2.5 mg · kg^−1^ for slow acetylators, 5 mg · kg^−1^ for intermediate acetylators, and 7.5 mg · kg^−1^ for fast acetylators, resulting in reduced adverse effects in fast acetylators while maintaining overall treatment efficacy in all acetylator phenotypes (63). In another clinical study, by using a regression model that considered patient NAT2 phenotype and body weight, Jung et al. retrospectively adapted INH dosages for slow and fast acetylators that would have maintained a desired plasma INH concentration range of 3.0 to 6.0 mg · liter^−1^ at 2 h postadministration (65).

The findings of the PBPK/PD analyses presented here are in concordance with these experimental findings, yet they extend the conclusions of those studies to administration schedules; patient characteristics, such as immune status; as well as the exposure to toxic metabolites. Hence, we propose that, on the basis of the findings of mechanistic modeling, rational adjustment of INH doses by consideration of the regional prevalence of NAT2 acetylator phenotypes (66) and affiliation with risk groups can increase overall treatment efficacy while simultaneously reducing the probability that the patient will experience toxic events, treatment failure, and the emergence of resistance in the future.

## Supplementary Material

Supplemental material
