# Supplementary material for: A Physiologically Based Pharmacokinetic Model of Isoniazid and Its Application in Individualizing Tuberculosis Chemotherapy
Source: Antimicrob Agents Chemother. 2016 Sep 23;60(10):6134–45. doi: 10.1128/AAC.00508-16 (PMC5038291; doi:10.1128/AAC.00508-16)
Supplement: Supplemental material [file supp_60_10_6134__index.html]

A Physiologically Based Pharmacokinetic Model of Isoniazid and Its Application in Individualizing Tuberculosis Chemotherapy — Supplemental material 

# A Physiologically Based Pharmacokinetic Model of Isoniazid and Its Application in Individualizing Tuberculosis Chemotherapy

## Supplemental material

- Supplemental file 1 -

  Additional description of model construction and validation, Table S1, and Figures S1 to S8.

  PDF, 1.3M
- Supplemental file 2 -

  Table S2: parameters used in population simulation

  XLSX, 832K
- Supplemental file 3 -

  Table S3: sampled beta for immune-deficient population

  XLSX, 38K
