# Supplementary material for: A Physiologically Based Pharmacokinetic Model of Isoniazid and Its Application in Individualizing Tuberculosis Chemotherapy
Source: Antimicrob Agents Chemother. 2016 Sep 23;60(10):6134–45. doi: 10.1128/AAC.00508-16 (PMC5038291; doi:10.1128/AAC.00508-16)
Supplement: Supplemental material [file AAC.00508-16_zac010165585so1.pdf]

Supplementary Material

PBPK Model Construction & Validation. Parameters of the physiologically-based pharmacokinetic (PBPK) models can be separated into 1) anthropometric parameters describing the anatomy and physiology of the human organism with its tissues, organs and sub-compartments (**Fig. SI 1**), 2) physiochemical properties (e.g. lipophilicity coefficient or the fraction of unbound drug) of the modeled compounds used to describe passive absorption and diffusion processes, and 3) kinetic parameters characterizing active transport and enzymatic reaction processes. In this study anthropometric parameters where taken form the corresponding studies. If the studies did not provide these parameters, mean patients provided by the PBPK modeling software (PK-Sim®; Version 6.0.3; Bayer Technology Services GmbH, Leverkusen, Germany) were used. The physicochemical parameters for INH and its metabolites were calculated with cheminformatics software (MarvinSketch; Version 15.11.30.0; ChemAxon Kft., Budapest, Hungary). Metabolic reactions and active transport were integrated into the PBPK model. Michaelis-Menten kinetics were used to describe the reaction rates. Tissue specific relative enzyme and transporter abundances were quantified by gene expression data provided by the PBPK modeling software (1).

Since each of the considered metabolites is found in the urine (2, 3), renal excretion processes for these all metabolites were integrated into the PBPK model. For acetylisoniazid, isonicotinic acid, isonicotinoyl glycine, hydrazine, acetylhydrazine, and diacetylhydrazine glomerular filtration was considered, while for isoniazid and acetylisoniazid an active tubular secretion process based on Michaelis-Menten kinetics was introduced to match experimental data (4). The INH conjugation reaction with α-

ketoglutarate and pyruvate were lumped together into the active tubular secretion process, since no pharmacokinetic data for those metabolites was available. Intravenous and oral administration protocols were adapted from the respective clinical study design. In the QD dosing regimens INH was administered every 24 h, while the BID dosing regimens consisted of half the cumulated daily INH doses administered every 12 h.

PD Model Construction & Validation.

Clinical data describing the early bacterial activity (EBA) in sputum of NAT2 phenotype-specific tuberculosis patients following a QD dosing regimen in the first two days of INH monotherapy (5, 6) was used for parameter identification of the PBPK/PD model. To limit the potentially misleading effect of outliers in the experimental data, only patient subgroups with more than three individuals were considered. The sampling patterns used in the original studies were adapted in the PBPK/PD simulations. The simulated difference in bacterial counts on the first day (0h < t < 24h) and on the second day (24h < t < 48h) of treatment were averaged to calculate the final EBA for each INH dose.

The pharmacodynamic (PD) model (**A1**) describes the change in mycobacterial counts in immune competent humans ($\mu(MT)_{IC}^{human}$) as sum of mycobacterial growth rate in immune deficient humans ($\mu(MT)_{ID}^{human}$), immune system dependent antimycobacterial killing (β^{human}) and INH dependent killing rate ($\gamma(INH)$). We conducted a literature review in order to identify *M. tuberculosis* growth rates in humans and mice*.* For untreated immune competent humans ($\mu(MT)_{IC}^{human}$) we found an averaged the growth rate of 0.0209 $\log_{10}$CFU·day^{-1} (7, 8, 5, 9, 10, 6, 11). To estimate β^{human} we used experimental derived *M. tuberculosis* growth rates in mice and immune deficient humans ($\mu(MT)_{ID}^{human}$). In immune competent mice, the mycobacterial growth rate was estimated

as 0.1355 $\log_{10}$CFU·day^{-1} ($\mu(MT)_{IC}^{mice}$) (12, 13) and 0.295 $\log_{10}$CFU·day^{-1} for immune deficient mice ($\mu(MT)_{ID}^{mice}$) (12), resulting in 0.1595 $\log_{10}$CFU·day^{-1} for β^{mice}. We assumed a constant ratio between the untreated immune dependent and immune deficient growth rates of *M. tuberculosis* in human and mice. From **A2**, we then calculated $\mu(MT)_{ID}^{human}$ as 0.0428 $\log_{10}$CFU·day^{-1} and from **A3** β^{human} as 0.0219 $\log_{10}$CFU·day^{-1}.

$$\mu(MT)_{IC}^{human} = \mu(MT)_{ID}^{human} - \beta^{human} - \gamma(INH) \qquad \textbf{A1}$$

$$\mu(MT)_{ID}^{human} = \frac{\mu(MT)_{IC}^{human} * \mu(MT)_{ID}^{mice}}{\mu(MT)_{IC}^{mice}} \qquad \textbf{A2}$$

$$\beta^{human} = \mu(MT)_{ID}^{human} - \mu(MT)_{IC}^{human} \qquad \textbf{A3}$$

SI Tables

Table S 1: PBPK model parameters for urinary excretion of INH and its metabolites

**Table S 1:** PBPK model parameters for urinary excretion of INH and its metabolites			
Renal excretion	V_{max}* (µmol·L^{-1}·min^{-1})	Km* (µmol·L^{-1})	Glomerular filtration rate
Isoniazid	20	300	0
Acetylisoniazid	0.3	20	0
Hydrazine	0	0	1
Diacetylhydrazine	0	0	0.4
Acetylisoniazid	0	0	1
Acetylhydrazine	0	0	0.2
Isonicotinic acid	0	0	1
Isonicotinoyl glycine	0	0	1

* Kinetic parameters for tubular secretion

Table S 2: Parameters used in population simulation

PatientPopulation.xlsx

Table S 3: Sampled beta for immune deficient population

immunde_deficient_beta.xlsx

SI Figures

Fig. SI 1: PBPK model structure in PK-Sim®.

Fig. SI 2: Simulated (lines) and experimental (symbols) PK profiles of A) INH (solid; circles) following a single 300 mg INH intravenous administration in fast acetylators (14), B) INH (solid; circles), acetylisoniazid (AcINH) (dashed; triangles), isonicotinic acid (INA) (dotted; squares), and isonicotinoyl glycine (INAG) (dash-dotted; diamonds) following a single 300 mg intravenous administration in fast acetylators (15). C) observed (2, 3) vs. predicted INH, AcINH, INA, and INAG plasma concentrations in fast acetylators following single intravenous INH dose of 300 mg.

Fig. SI 3: Simulated (lines) and experimental (circles) (16) INH PK profiles A) following a single 300 mg oral INH dose in intermediate (FS) acetylators (solid), B) INH PK profiles following single a 300 mg, 600 mg, or 900 mg oral INH dose in fast (FF) acetylators (dashed). C) observed (16) vs. predicted INH plasma concentrations for intermediate and fast acetylators following a single oral INH dose of 300 mg, and 300 mg, 600 mg, or 900 mg, respectively.

Fig. SI 4: Simulated INH A-C) and AcINH D-E) pharmacokinetic profiles of virtual patient populations comprising 1,000 individuals. The population median (solid), the 25th and 75th (dashed), and 5th and 95th (dotted) percentiles after receiving a single oral dose of 300 mg INH are shown. Subfigure A) and D) show slow, B) and E) intermediate, and C) and F) fast acetylator populations.

Fig. SI 5: Plasma concentration profiles of INH and its metabolites as simulations (lines) and experimental data (symbols) (17). A) PK profiles of slow acetylators (SS) for INH (black solid, circles), acetylhydrazine (AcHz) (black solid, triangles), and intermediate

acetylators (FS) for INH (grey dashed, circles), AcHz (grey dashed, triangles). B) observed (17) vs. predicted INH and AcHz plasma concentrations for slow and intermediate acetylators following single oral INH dose of 300 mg.

Fig. SI 6: Plasma concentration profiles of INH and its metabolites as simulations (lines) and experimental data (symbols) (18). A) PK profiles of slow acetylators (SS) for INH (black solid, circles) and hydrazine (Hz) (black solid, right-pointing triangles) and intermediate acetylators (FS) for INH (grey dashed, circles) and Hz (grey dashed, right-pointing triangles). B) observed (18) vs. predicted INH and Hz plasma concentrations for slow and intermediate acetylators following single oral INH dose of 300 mg.

Fig. SI 7: Plasma concentration profiles of INH and its metabolites as simulations (lines) and experimental data (symbols) (19). A) PK profiles of slow acetylators (SS) for AcHz (black solid, left-pointing triangles), diacetylhydrazine (DiAcHz) (black solid, down-pointing triangles) and intermediate acetylators (FS) for AcHz (grey dashed, left-pointing triangles), DiAcHz (grey dashed, down-pointing triangles). B) observed (19) vs. predicted AcHz and DiAcHz plasma concentrations for slow and intermediate acetylators following single oral INH dose of 300 mg.

Fig. SI 8: Plasma concentration profiles of INH and its metabolites as simulations (lines) and experimental data (symbols) (20). A) PK profiles of slow acetylators (SS) for INH (black solid, circles), AcINH (black solid, squares), AcHz (black solid, left-pointing triangles), DiAcHz (black solid, down-pointing triangles) and fast acetylators (FF) for INH (grey dashed, circles), AcINH (grey dashed, squares), AcHz (grey dashed, left-pointing triangles), DiAcHz (grey dashed, down-pointing triangles) and intermediate acetylators (FS) for INH (grey dotted, circles), AcINH (grey dotted, squares), AcHz (grey dotted, left-

pointing triangles), DiAcHz (grey dotted, down-pointing triangles). B) observed (20) vs. predicted INH, AcINH, AcHz, and DiAcHz plasma concentrations for slow, intermediate, and fast acetylators following single oral INH dose of 300 mg.

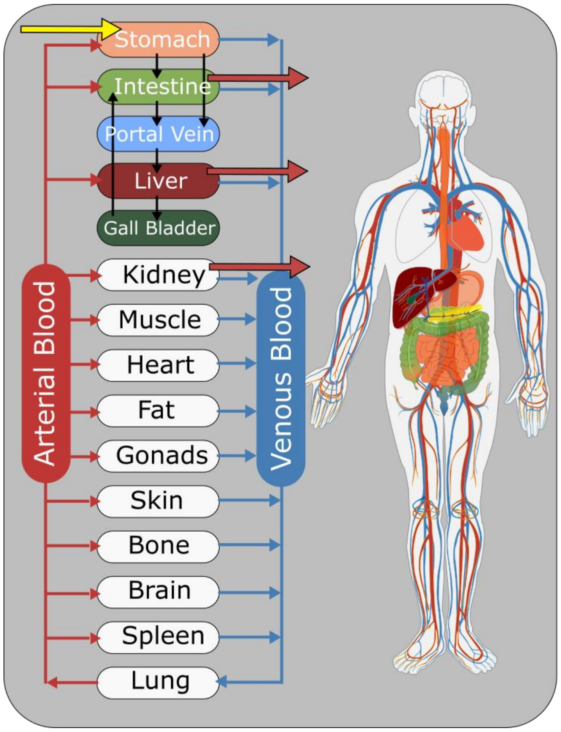

Fig. SI 1: PBPK model structure in PK-Sim®.

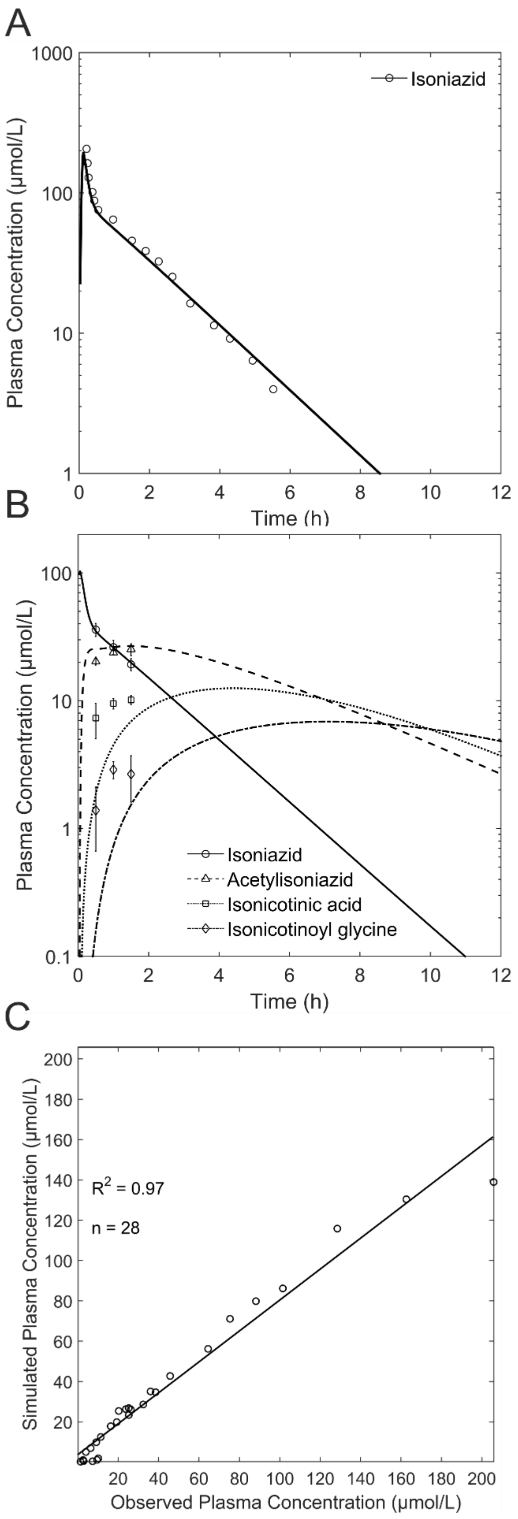

Fig. SI 2: Simulated (lines) and experimental (symbols) PK profiles of A) INH (solid; circles) following a single 300 mg INH intravenous administration in fast acetylators (14), B) INH (solid; circles), acetylisoniazid (AcINH) (dashed; triangles), isonicotinic acid (INA) (dotted; squares), and isonicotinoyl glycine (INAG) (dash-dotted; diamonds) following a single 300 mg intravenous administration in fast acetylators (15). C) Observed (2, 3) vs. predicted INH, AcINH, INA, and INAG plasma concentrations in fast acetylators following single intravenous INH dose of 300 mg.

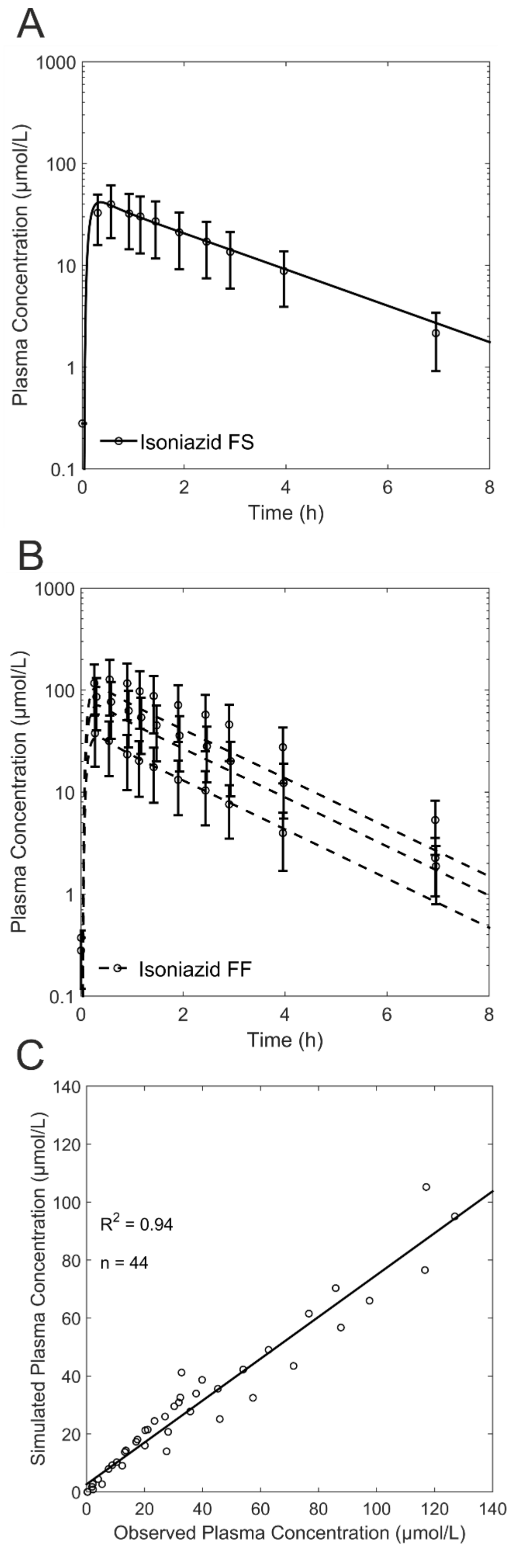

Fig. SI 3: Simulated (lines) and experimental (circles) (16) INH PK profiles A) following a single 300 mg oral INH dose in intermediate (FS) acetylators (solid), B) INH PK profiles following single a 300 mg, 600 mg, or 900 mg oral INH dose in fast (FF) acetylators (dashed). C) Observed (16) vs. predicted INH plasma concentrations for intermediate and fast acetylators following a single oral INH dose of 300 mg, and 300 mg, 600 mg, or 900 mg, respectively.

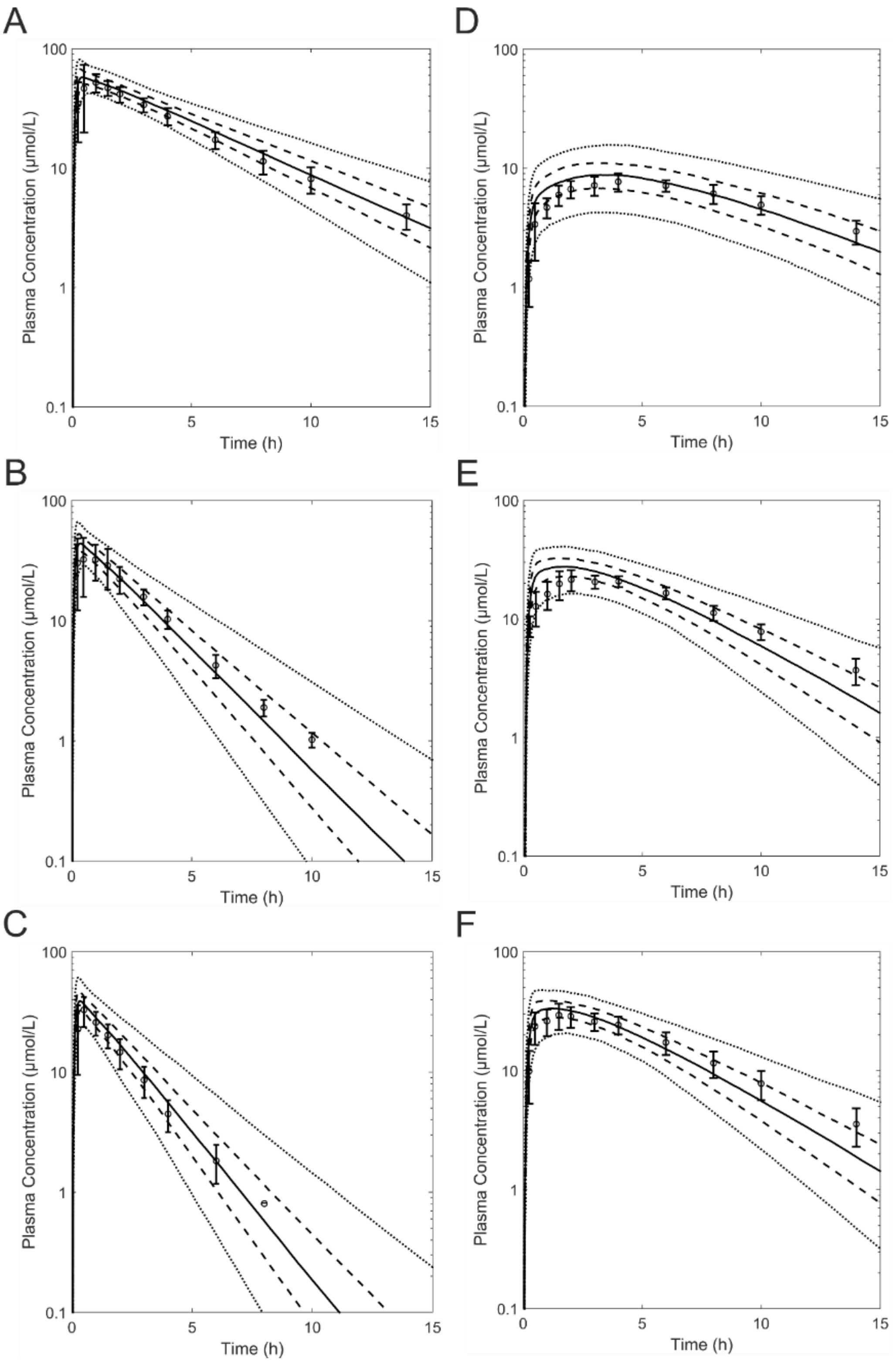

Fig. SI 4: Simulated INH A-C) and AcINH D-E) pharmacokinetic profiles of virtual patient populations comprising 1,000 individuals. The population median (solid), the 25th and 75th (dashed), and 5th and 95th (dotted) percentiles after receiving a single oral dose of 300 mg INH are shown. Subfigure A) and D) show slow, B) and E) intermediate, and C) and F) fast acetylator populations.

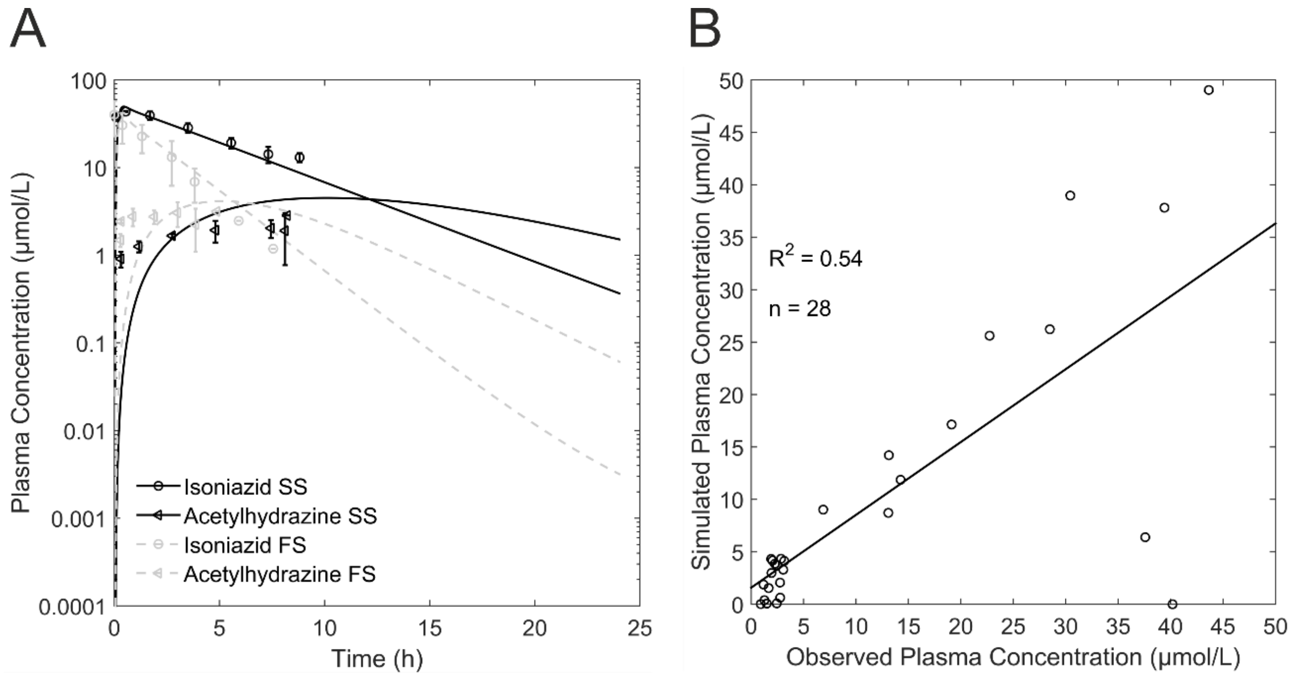

Fig. SI 5: Plasma concentration profiles of INH and its metabolites as simulations (lines) and experimental data (symbols) (17). A) PK profiles of slow acetylators (SS) for INH (black solid, circles), acetylhydrazine (AcHz) (black solid, triangles), and intermediate acetylators (FS) for INH (grey dashed, circles), AcHz (grey dashed, triangles). B) Observed (17) vs. predicted INH and AcHz plasma concentrations for slow and intermediate acetylators following single oral INH dose of 300 mg.

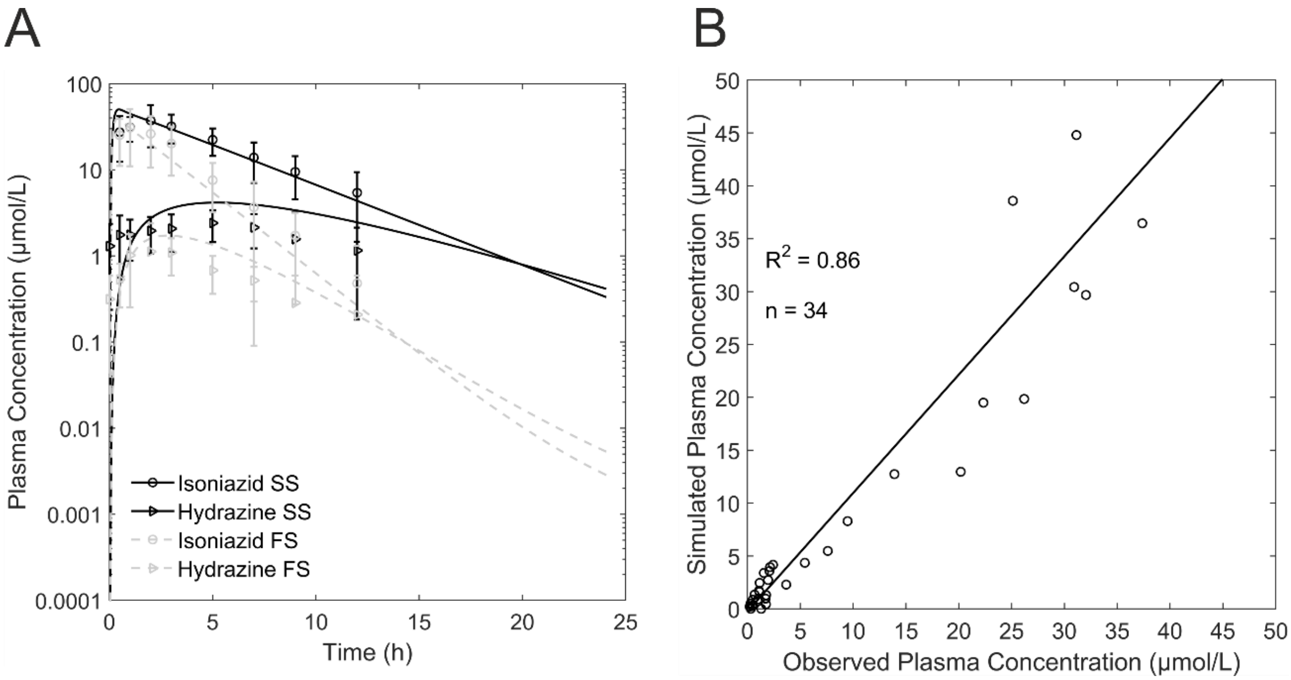

Fig. SI 6: Plasma concentration profiles of INH and its metabolites as simulations (lines) and experimental data (symbols) (18). A) PK profiles of slow acetylators (SS) for INH (black solid, circles) and hydrazine (Hz) (black solid, right-pointing triangles) and intermediate acetylators (FS) for INH (grey dashed, circles) and Hz (grey dashed, right-pointing triangles). B) Observed (18) vs. predicted INH and Hz plasma concentrations for slow and intermediate acetylators following single oral INH dose of 300 mg.

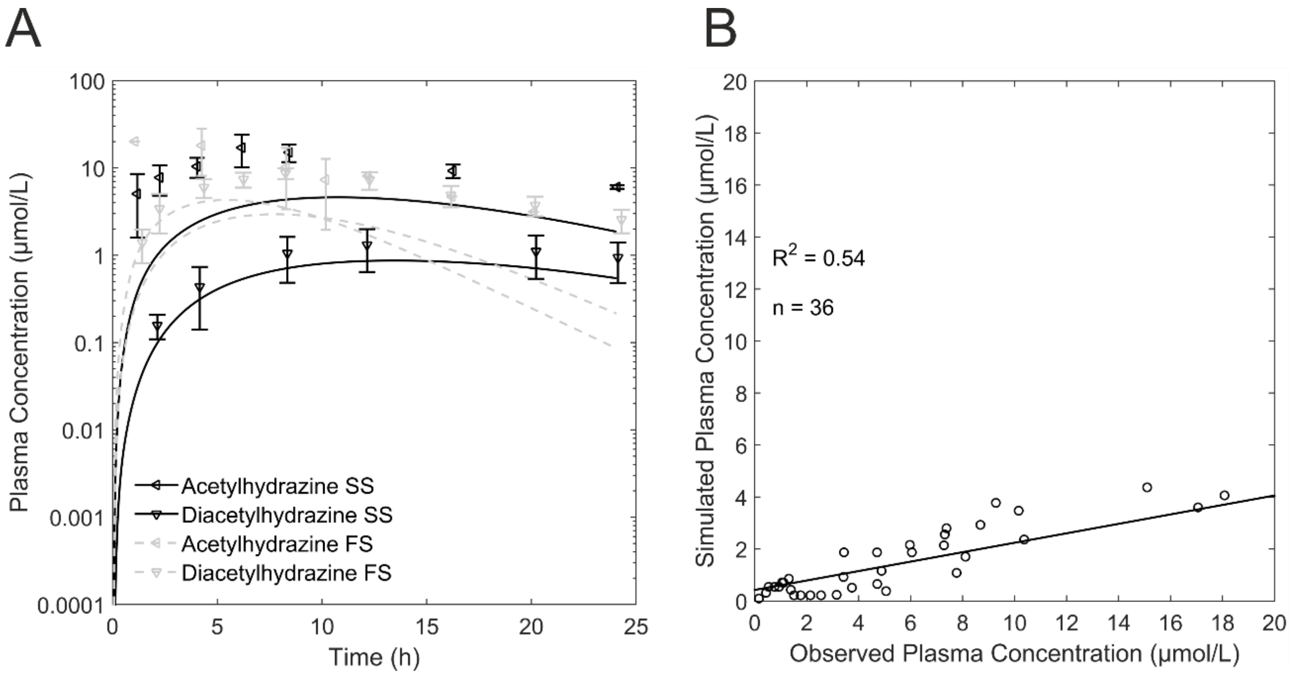

Fig. SI 7: Plasma concentration profiles of INH and its metabolites as simulations (lines) and experimental data (symbols) (19). A) PK profiles of slow acetylators (SS) for AcHz (black solid, left-pointing triangles), diacetylhydrazine (DiAcHz) (black solid, down-pointing triangles) and intermediate acetylators (FS) for AcHz (grey dashed, left-pointing triangles), DiAcHz (grey dashed, down-pointing triangles). B) Observed (19) vs. predicted AcHz and DiAcHz plasma concentrations for slow and intermediate acetylators following single oral INH dose of 300 mg.

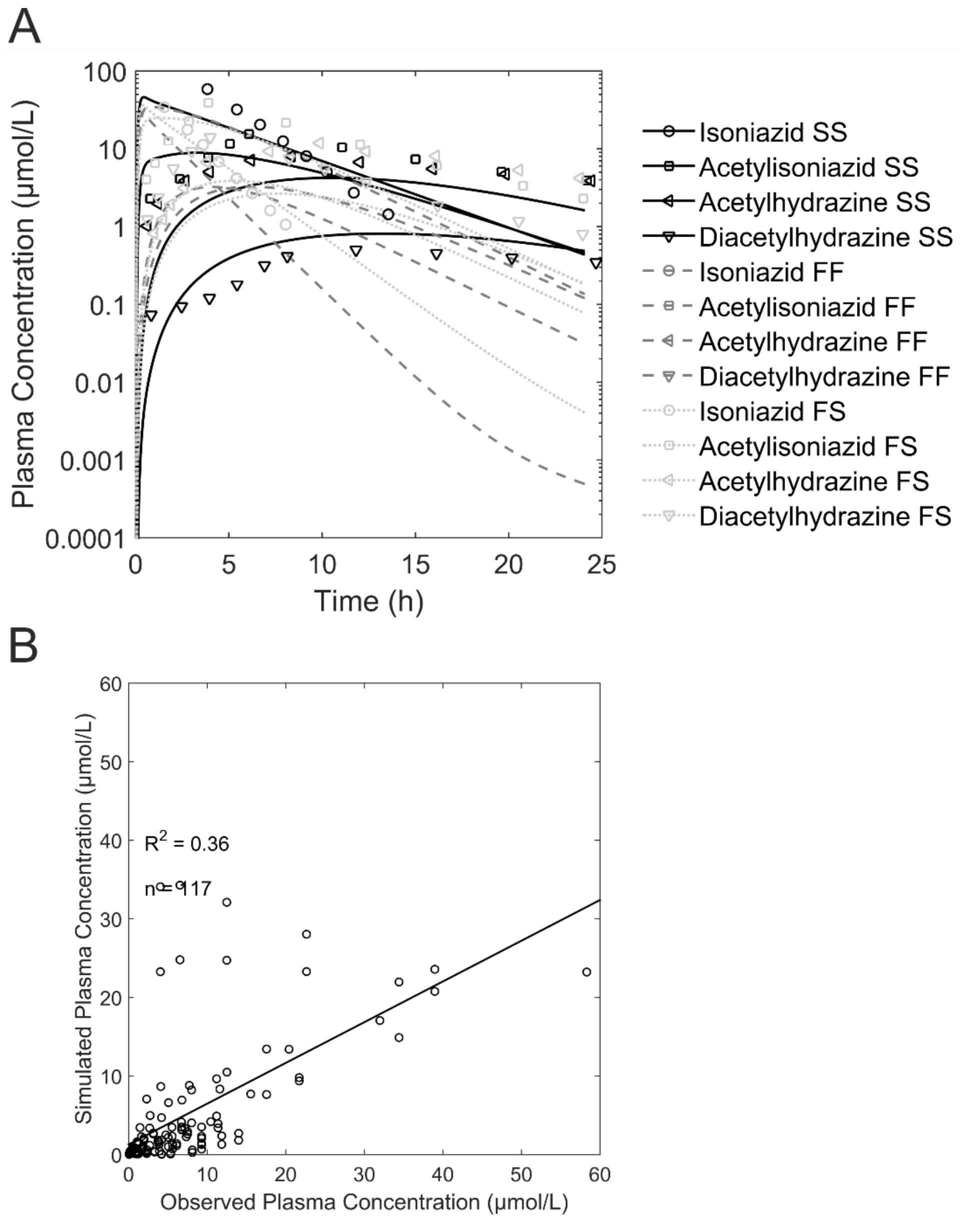

Fig. SI 8: Plasma concentration profiles of INH and its metabolites as simulations (lines) and experimental data (symbols) (20). A) PK profiles of slow acetylators (SS) for INH (black solid, circles), AcINH (black solid, squares), AcHz (black solid, left-pointing triangles), DiAcHz (black solid, down-pointing triangles) and fast acetylators (FF) for INH (grey dashed, circles), AcINH (grey dashed, squares), AcHz (grey dashed, left-pointing triangles), DiAcHz (grey dashed, down-pointing triangles) and intermediate acetylators (FS) for INH (grey dotted, circles), AcINH (grey dotted, squares), AcHz (grey dotted, left-pointing triangles), DiAcHz (grey dotted, down-pointing triangles). B) Observed (20) vs. predicted INH, AcINH, AcHz, and DiAcHz plasma concentrations for slow, intermediate, and fast acetylators following single oral INH dose of 300 mg.

SI References

1. **Meyer M**, **Schneckener S**, **Ludewig B**, **Kuepfer L**, **Lippert J**. 2012. Using expression data for quantification of active processes in physiologically based pharmacokinetic modeling. Drug Metab Dispos **40**:892–901.

2. **Mitchell JR**, **Thorgeirsson UP**, **Black M**, **Timbrell JA**, **Snodgrass WR**, **Potter WZ**, **Jollow HR**, **Keiser HR**. 1975. Increased incidence of isoniazid hepatitis in rapid acetylators: possible relation to hydranize metabolites. Clin Pharmacol Ther **18**:70–79.

3. **Ellard G a**, **Gammon PT**. 1976. Pharmacokinetics of isoniazid metabolism in man. J Pharmacokinet Biopharm **4**:83–113.

4. **Mitchell JR**, **Thorgeirsson UP**, **Black M**, **Timbrell JA**, **Snodgrass WR**, **Potter WZ**, **Jollow HR**, **Keiser HR**. 1975. Increased incidence of isoniazid hepatitis in rapid acetylators: possible relation to hydranize metabolites. Clin Pharmacol Ther **18**:70–9.

5. **Donald PR**, **Sirgel FA**, **Botha FJ**, **Seifart HI**, **Parkin DP**, **Vandenplas ML**, **Van de Wal BW**, **Maritz JS**, **Mitchison DA**. 1997. The early bactericidal activity of isoniazid related to its dose size in pulmonary tuberculosis. Am J Respir Crit Care Med **156**:895–900.

6. **Donald PR**, **Sirgel F a**, **Venter A**, **Parkin DP**, **Seifart HI**, **van de Wal BW**, **Werely C**, **van Helden PD**, **Maritz JS**. 2004. The Influence of Human N-Acetyltransferase Genotype on the Early Bactericidal Activity of Isoniazid. Clin Infect Dis **39**:1425–1430.

7. **Jindani A**, **Aber VR**, **Edwards EA**, **Mitchison DA**. 1980. The early bactericidal activity of drugs in patients with pulmonary tuberculosis. Am Rev Respir Dis **121**:939–49.

8. **Sirgel FA**, **Botha FJ**, **Parkin DP**, **Van De Wal BW**, **Donald PR**, **Clark PK**, **Mitchison DA**. 1993. The early bactericidal activity of rifabutin in patients with pulmonary tuberculosis measured by sputum viable counts: a new method of drug assessment. J Antimicrob Chemother **32**:867–75.

9. **Donald PR**, **Sirgel FA**, **Venter A**, **Parkin DP**, **Van de Wal BW**, **Barendse A**, **Smit E**, **Carman D**, **Talent J**, **Maritz J**. 2001. Early bactericidal activity of amoxicillin in combination with clavulanic acid in patients with sputum smear-positive pulmonary tuberculosis. Scand J Infect Dis **33**:466–9.

10. **Donald PR**, **Sirgel FA**, **Venter A**, **Smit E**, **Parkin DP**, **Van de Wal BW**, **Doré CJ**, **Mitchison DA**. 2002. The early bactericidal activity of streptomycin. Int J Tuberc Lung Dis **6**:693–8.

11. **Sirgel FA**, **Donald PR**, **Odhiambo J**, **Githui W**, **Umapathy KC**, **Paramasivan CN**, **Tam CM**, **Kam KM**, **Lam CW**, **Sole KM**, **Mitchison DA**. 2000. A multicentre study of the early bactericidal activity of anti-tuberculosis drugs. J Antimicrob Chemother **45**:859–70.

12. **McKinney JD**, **Höner zu Bentrup K**, **Muñoz-Elías EJ**, **Miczak A**, **Chen B**, **Chan WT**, **Swenson D**, **Sacchettini JC**, **Jacobs WR**, **Russell DG**. 2000. Persistence of Mycobacterium tuberculosis in macrophages and mice requires the glyoxylate shunt enzyme isocitrate lyase. Nature **406**:735–8.

13. **Dubnau E**, **Chan J**, **Mohan VP**, **Smith I**. 2005. Responses of Mycobacterium tuberculosis to Growth in the Mouse Lung. Infect Immun **73**:3754–3757.

14. **Boxenbaum HG**, **Riegelman S**, **Elashoff RM**. 1974. Statistical estimations in pharmacokinetics. J Pharmacokinet Biopharm **2**:123–48.

15. **Ellard G a**, **Gammon PT**, **Wallace SM**. 1972. The determination of isoniazid and its metabolites acetylisoniazid, monoacetylhydrazine, diacetylhydrazine, isonicotinic acid and isonicotinylglycine in serum and urine. Biochem J **126**:449–458.

16. **Kubota R**, **Ohno M**, **Hasunuma T**, **Iijima H**, **Azuma J**. 2007. Dose-escalation study of isoniazid in healthy volunteers with the rapid acetylator genotype of arylamine N-acetyltransferase 2. Eur J Clin Pharmacol **63**:927–933.

17. **Peretti E**, **Karlaganis G**, **Lauterburg BH**. 1987. Acetylation of acetylhydrazine, the toxic metabolite of isoniazid, in humans. Inhibition by concomitant administration of isoniazid. J Pharmacol Exp Ther **243**:686–9.

18. **Pea F**, **Milaneschi R**, **Baraldo M**, **Talmassons G**, **Furlanut M**. 1999. Isoniazid and its Hydrazine Metabolite in Patients with Tuberculosis. Clin Drug Investig **17**:145–154.

19. **Lauterburg BH**, **Smith C V**, **Todd EL**, **Mitchell JR**. 1985. Pharmacokinetics of the toxic hydrazino metabolites formed from isoniazid in humans. J Pharmacol Exp Ther **235**:566–570.

20. **Lauterburg BH**, **Smith C V.**, **Mitchell JR**. 1981. Determination of isoniazid and its hydrazino metabolites, acetylisoniazid, acetylhydrazine, and diacetylhydrazine in

human plasma by gas chromatography—mass spectrometry. J Chromatogr B Biomed Sci Appl.
